# Temporal succession of microbiomes and resistomes during buried rat carcass decomposition

**DOI:** 10.1128/msphere.00437-26

**Published:** 2026-08-12

**Authors:** Daijing Yu, Liuyaoxing Zhang, Damu Agu, Niu Gao, Yuntian Xiao, Meng Zhang, Jun Zhang, Jiangwei Yan

**Affiliations:** 1 School of Forensic Medicine, Shanxi Medical University 74648, Taiyuan, Shanxi, China; 2 Shanxi Key Laboratory of Forensic Medicine, Jinzhong, Shanxi, China; 3 Shanxi Province Engineering Research Center of Forensic Identification, Taiyuan, Shanxi, China; 4 MOE Key Laboratory of Coal Environmental Pathogenicity and Prevention, Jinzhong, Shanxi, China; University of Nebraska Medical Center College of Medicine, Omaha, Nebraska, USA

**Keywords:** buried carcass decomposition, microbial community, antibiotic resistance genes (ARGs), mobile genetic elements (MGEs), horizontal gene transfer (HGT)

## Abstract

Burial is a prevalent approach for disposing of human and animal carcass. Carcass decomposition is a key natural disturbance that reshapes microbial communities and modulates biogeochemical cycles. Animal intestines serve as critical natural reservoirs of antibiotic resistance genes (ARGs), and carcass decomposition concurrently remodels intestinal bacterial assemblages and resistome profiles. Most previous studies have characterized microbial succession in surface-exposed carcasses, yet the coordinated temporal shifts of intestinal microbiota, resistomes, and horizontal gene transfer (HGT) signatures within buried carcasses remain underexplored. We established a buried rat carcass model and performed metagenomic sequencing to characterize temporal dynamics of intestinal bacteria, ARGs, mobile genetic elements (MGEs), and HGT events. Bacterial communities underwent directional succession accompanied by reduced α-diversity and stage-specific β-diversity. Proteobacteria gradually outcompeted Firmicutes and Bacteroidetes to become dominant taxa. The resistome followed regular temporal changes: tetracycline and macrolide-lincosamide-streptogramin (MLS) ARGs prevailed in early decomposition, while multidrug, β-lactam, polymyxin, and quinolone ARGs accumulated in mid-late stages, with ARG richness peaking on day 28. Approximately 83% of temporally dynamic ARG subtypes were positively correlated with decomposition duration. Procrustes analysis (*R*^2^ = 0.859) revealed strong correlations between bacterial succession and resistome dynamics, with Proteobacteria as potential multidrug-resistant hosts and major HGT donors. This study identifies a correlative cascade of microbial succession that drives MGE functional shift, which in turn increased HGT potential and ultimately leads to resistome accumulation in buried carcass intestinal habitats. These findings expand the theoretical framework of disturbance-driven microbial-resistome co-succession and offer insights into drivers of antibiotic resistance propagation in terrestrial cadaver systems.

**IMPORTANCE:**

Animal carcass burial is a ubiquitous natural terrestrial disturbance, and carcass intestinal contents represent a major endogenous reservoir of environmental antibiotic resistance genes (ARGs). Although extensive research has characterized microbial succession of exposed carcasses, the co-occurrence patterns of gut microbiome and resistome together with associated horizontal gene transfer (HGT) under buried conditions remain underexplored. Using metagenomic profiling of decomposing rat intestinal feces, this study explores a potential correlative cascade spanning bacterial succession, mobile genetic element (MGE) functional shifts, increased HGT potential, and gradual ARG enrichment. Our findings expand the ecological data set focused on subsurface buried carcass habitats and advance mechanistic knowledge of coupled microbiome-resistome succession driven by postmortem decomposition.

## INTRODUCTION

Ecological succession is a central research focus in community ecology, providing a framework to predict how ecosystem diversity and function respond to external disturbances such as animal carcass decomposition (1). Disturbance-driven community succession often exhibits patterns of convergence, divergence, or non-linear gradual change, with trajectories and directions shaped jointly by ecosystem type, environmental conditions, disturbance intensity, and spatiotemporal scales (2–4). Elucidating the succession patterns of microbial communities under disturbance is essential for understanding biogeochemical cycling, community assembly, and the maintenance of ecological functional stability. Animal carcass decomposition represents a typical and intensive natural disturbance in terrestrial ecosystems, driving biogeochemical cycles and reshaping microbial community structure. Therefore, systematically analyzing carcass decomposition-mediated microbial succession contributes to the theoretical foundation of community assembly in disturbed ecosystems. Burial, as a globally prevalent method of corpse disposal, creates distinct subsurface microenvironments that drive decomposition processes, and microbial succession patterns may differ from those of exposed carcasses.

Carcass decomposition is a key process in terrestrial biogeochemical cycling. A single carcass releases approximately 20% of its carbon and 2%–3% of its nitrogen into the surrounding environment, and terrestrial ecosystems accumulate an average of about 5,000 kg of carcass biomass per square kilometer per year (5, 6). Microorganisms are the primary drivers of decomposition, with nearly 80% of animal carcasses being degraded by the synergistic action of microbes and insects (7, 8). After death, the host immune barrier collapses, metabolism ceases, and cell autolysis begins, releasing large amounts of intracellular nutrients and macromolecular organic substrate, thereby providing the substrate basis and a suitable microhabitat for microbial proliferation and migration (8–10).

Carcass decomposition is a highly dynamic, stage-specific successional process. Nutrient availability, redox conditions, and pH in the microhabitat change continuously as decomposition progresses, creating sustained environmental filtering pressures that drive directional community turnover and adaptive shifts (5). In the early stage, the host’s intestinal anaerobes, freed from immune restriction, proliferate extensively and dominate early organic substrates degradation (8, 11). The accumulation of putrefactive gases leading to abdominal rupture is a critical turning point: the gut environment shifts from anaerobic to aerobic, and the accumulation of endogenous ammonia causes a marked increase in pH, resulting in a decline of anaerobic taxa such as *Lactobacillaceae* and *Bacteroidaceae,* and the gradual enrichment of oxygen-tolerant groups including Proteobacteria (12, 13). Multiple animal model studies have confirmed that the microbial succession during carcass decomposition follows predictable temporal patterns: early enrichment of Proteobacteria, late dominance of Actinobacteria, and a continuous decline in community diversity (14). Existing research has largely focused on changes in community structure, neglecting the dynamics of functional genes during decomposition.

On the basis of microbial community temporal succession, antibiotic resistance genes (ARGs), as a class of key functional genes, represent a critical yet easily overlooked ecological process with significant environmental risks. Animal gut microbiota is considered an important reservoir of environmental ARGs, and a wide range of resistant subtypes are commonly enriched in the intestines of livestock and wild animals (15–18). After death, the lysis of intestinal bacteria releases intracellular ARGs together with organic matter, cell debris, and genetic material into the surrounding environment, greatly increasing the abundance and diversity of environmental ARGs and making carcass leachate a major point source for the proliferation of resistant bacteria and the dissemination of resistance genes (19, 20). As emerging persistent contaminants, ARGs can spread across strains and species through horizontal gene transfer (HGT); if such genes are transferred to environmental pathogens, they may threaten the efficacy of clinical antibiotic treatment and the stability of terrestrial ecosystems (21).

Mobile genetic elements (MGEs), including plasmids, integrons, and transposons, facilitate the capture, accumulation, and horizontal spread of resistance genes among pathogenic and commensal bacteria, making them key vectors for ARG dissemination (22, 23). They also serve as a vital link between bacterial community succession and resistome dynamics. Studies have shown that community turnover, cell lysis, and microenvironmental stress can alter MGE functions, thereby increasing the frequency of HGT and subsequently driving ARG enrichment and turnover (24–26). Although previous work has elucidated the patterns of microbial succession during carcass decomposition in open-air systems, most studies have focused on surface soil environments and have been limited to community structure and diversity, with few targeting the gut as a core ARG reservoir under burial conditions. More importantly, the cascading relationships among intestinal microbial succession, MGE functional shifts, HGT potential fluctuations, and resistome remodeling during decomposition remain poorly understood. The co-succession patterns of fecal microbiota, ARGs, and MGEs under buried conditions are still unclear.

In this study, we established a rat carcass decomposition model in a realistic field burial environment and employed metagenomic sequencing to systematically investigate the temporal dynamics of bacterial communities, ARGs, and MGEs during decomposition, focusing on intestinal feces as the core ARG reservoir. The specific scientific questions addressed are as follows: (i) the temporal succession trajectories and diversity changes of intestinal bacterial communities under burial conditions; (ii) the dynamic changes of ARGs and MGEs at different decomposition stages; and (iii) the potential link between bacterial succession and resistome composition, as well as the characteristics of MGE-mediated HGT at key decomposition stages. This study aims to elucidate the co-succession patterns of gut microbiota and the resistome during buried carcass decomposition, to improve the theoretical framework of resistance gene dissemination under natural disturbance, and to provide fundamental data and a theoretical basis for understanding endogenous resistance evolution driven by carcass degradation in terrestrial ecosystems.

## MATERIALS AND METHODS

### Experimental design and sample collection

The experimental design and sample collection methods employed in this study were consistent with those previously published by our study (27). An overview is provided below. Forty-eight rats were obtained from the Experimental Animal Center of Shanxi Medical University. All rats were euthanized by cervical dislocation, and the carcasses were buried in homogeneous soil (approximately 20 cm deep) to minimize interference from insects and scavengers. This study adhered to the ARRIVE guidelines and relevant ethical provisions of the UK Animals (Scientific Procedures) Act 1986 and was approved by the Shanxi Medical University Committee for the Management and Use of Laboratory Animals (Approval No. 2023-064). On days 0, 1, 3, 7, 14, 21, 28, and 35 post-burial, six carcasses (equal numbers of males and females) were exhumed at each time point. The carcasses were transported to the laboratory under refrigerated conditions, where rectal contents were aseptically dissected and collected as fecal samples. A total of 48 samples were collected and stored at −80°C until subsequent processing.

### DNA extraction and metagenomic sequencing

DNA extraction, library preparation, and metagenomic sequencing procedures followed methods previously published by the study (27). Total DNA was extracted from fecal samples using the E.Z.N.A. Viral DNA Kit (Omega Bio-tek, Norcross, GA, USA) according to the manufacturer’s instructions. DNA quality was assessed spectrophotometrically, and only high-quality samples (OD_260/280_ = 1.8–2.2; OD_260/230_ ≥ 2.0) were selected for downstream library preparation. Metagenomic libraries were constructed using the TruSeq Nano DNA Sample Preparation Kit (Illumina, San Diego, CA, USA) with 1 µg of total DNA used as input. The library preparation process included DNA end repair, A-tailing, and ligation of indexed adapters, as described in the Illumina protocol. Libraries were size selected for target fragments of approximately 400 bp using 2% Low Range Ultra Agarose gel electrophoresis and were subsequently amplified by PCR using Phusion DNA polymerase (NEB, Rosemont, IL, USA) for 15 cycles. Sequencing was performed on an Illumina NovaSeq platform using a paired-end 150 bp (PE150) configuration by Shanghai Biozeron Biotechnology Co., Ltd. (Shanghai, China).

### Bioinformatics analysis

### Sequence quality control, assembly, and gene quantification

Raw paired-end sequencing reads underwent quality control using Trimmomatic (version 0.40, https://github.com/usadellab/Trimmomatic) with the parameters SLIDINGWINDOW:4:15 and MINLEN:75 (28). To eliminate host contamination, reads were aligned against the rat reference genome using Bowtie2, and all successfully aligned reads (mRatBN7.2) were removed (29). High-quality reads underwent *de novo* assembly using MEGAHIT software (v1.2.9) with the parameter --min-contig-len 1000 (30). Assembly quality was assessed using QUAST (31). Open reading frames were predicted from contigs using Prodigal software (v2.6.3) in metagenomic mode (32). Predicted gene sequences were clustered to remove redundancy using CD-HIT-EST (similarity ≥95%, coverage ≥90%). Quantification of non-redundant genes was performed using Salmon (v1.8.0) in metagenomic mode, yielding TPM values and raw counts for each gene across all samples for subsequent analyses (33–35).

### Annotation of antibiotic resistance genes and mobile genetic elements

The online workflow ARGs-OAP 3.0 (36), integrated with the SARG database (v3.2.1-S), performs a comprehensive analysis of antibiotic resistance genes in paired-end sequencing reads. This workflow uses the stage_one.py and stage_two.py scripts to generate normalized abundance data (including cell-normalized results) across different classification levels (gene, subtype, and type) (36, 37).

Predicted protein sequences were aligned against the SARG database (v3.2.1) using BLASTX (e-value ≤10⁻⁷) (38) to identify antibiotic resistance genes. An ORF was annotated as an ARG if it met the following criteria: best-match similarity ≥70% with the reference sequence, alignment length ≥25 amino acids, and coverage ≥70%. Such open reading frames were annotated as antibiotic resistance gene sequences (39). Contigs harboring at least one ARG were defined as antibiotic resistance gene-carrying sequences (ACCs) (40). To annotate mobile genetic elements, all predicted protein sequences on these ACCs were aligned against the mobileOG-db 2.0 database using DIAMOND.

### Analysis of horizontal gene transfer events

Afterward, contigs were filtered to a minimum length of 2 kb; they were analyzed using WAAFLE (https://github.com/biobakery/waafle), which is integrated into BP-Tracer, to detect potential HGT in each contig. The HGT algorithm comprised two main steps: (i) BLAST was used to perform nucleotide-level searches of input contigs against a reference pangenome database via waafle_search.py. (ii) Taxonomic classification of contigs, including HGT identification, was performed using waafle_orgscorer.py. Only HGT events were retained for downstream analysis. The number of HGT events per contig was normalized by contig length (events per kb).

### Data processing and statistical analysis

All statistical analyses and visualizations were performed using R (v4.1.2; https://www.r-project.org). For bacterial community analysis, principal coordinate analysis (PCoA) based on Bray-Curtis distances was used to visualize β-diversity (vegan package). Prior to multivariate analysis, all abundance matrices were Hellinger-transformed to reduce the influence of low-abundance taxa. One-way ANOVA with Tukey’s honestly significant difference (HSD) *post hoc* test was applied to evaluate temporal differences in bacterial Shannon α-diversity. PERMANOVA and ANOSIM (41, 42) were performed to quantify the effect of decomposition time on bacterial community structure. For resistome profiling, ARG abundance was quantified as total copy numbers per cell for each type and subtype. Temporal dynamics of ARG abundance were assessed via Spearman correlation (ggpubr::stat_cor) (43).

To explore interactions between bacterial communities and resistome profiles, a co-occurrence network was constructed based on dominant bacterial genera (mean relative abundance >1%) and the top 50 high-copy-number ARG subtypes. Network structural characteristics were calculated and visualized using the igraph (44) and ggraph packages (45). Procrustes analysis and Mantel tests (46) were performed on Hellinger-standardized Bray-Curtis distance matrices to evaluate synchronous successional patterns among bacterial, ARG, and MGE community structures (vegan package). For MGE quantification, transcripts per million (TPM) normalization was applied to MGE annotations, and the relative proportion of each MGE functional category and carrier type was calculated based on total MGE TPM per sample. HGT events predicted by WAAFLE (see section 2.3.3) were counted per time point. Donor taxonomy at the phylum level was extracted from the TAXONOMY_A field in WAAFLE output. Spearman correlation was used to assess relationships between decomposition time, HGT frequency, MGE abundance, and ARG dynamics. All multiple comparisons were corrected using the Benjamini-Hochberg false discovery rate (FDR) method (47), and statistical significance was defined as FDR < 0.05.

## RESULTS

A total of 48 samples generated 1.87 billion high-quality paired-end reads, averaging 39.0 million reads (5.85 Gb) per sample. The mean Q20 and Q30 values were 98.99% and 96.96%, respectively, indicating high accuracy. *De novo* assembly using MEGAHIT yielded 1.73 million contigs (average 36,064 per sample), with a mean total assembled length of 129.5  Mb per sample and an average N50 of 8.2  kb. The longest contig reached 522  kb. Nonpareil curves confirmed sequencing depth saturation (Fig. S1).

**Fig 1:**
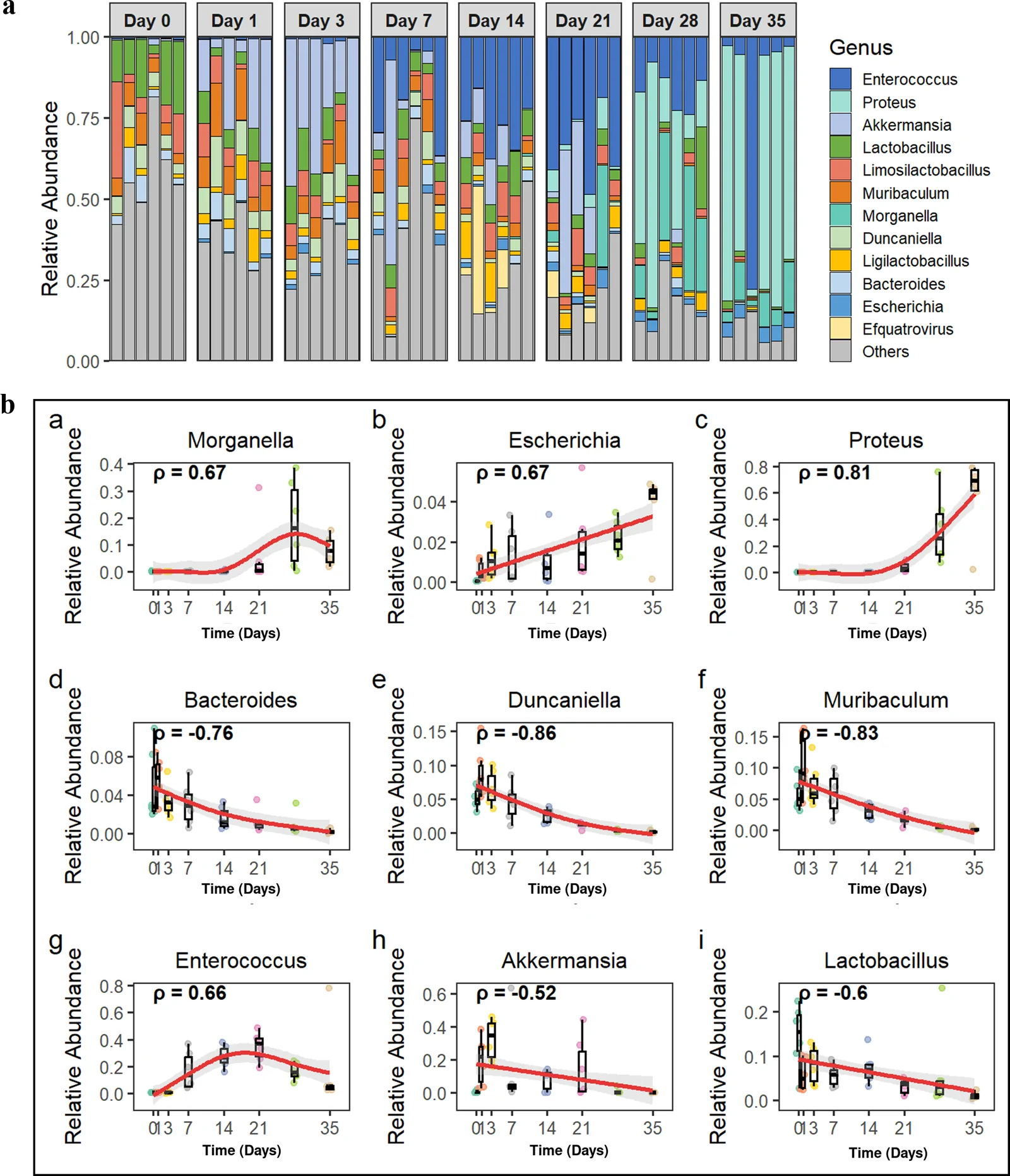
(**a**) Bacterial community composition at the genus level during different stages of carcass decomposition. Only those taxa with mean relative abundance more than 1% are shown. (**b**) Spearman correlation analysis of dominant bacterial genera with decomposition time. Relative abundance dynamics of dominant bacterial genera (mean relative abundance > 1%) significantly correlated with decomposition time (|ρ| > 0.5, *P* < 0.05).

### Bacterial community composition and succession during carcass decomposition

Carcass decomposition induced pronounced temporal turnover in bacterial community composition. At the phylum level, Firmicutes (37.36%), Proteobacteria (25.64%), Bacteroidetes (16.67%), and Verrucomicrobia (11.13%) dominated across all time points (Fig. S2). Proteobacteria increased continuously from 8.25% at day 0 to 76.20% at day 35, whereas Bacteroidetes declined from 28.50% to 1.05%. Firmicutes remained abundant in early and middle stages, while Verrucomicrobia peaked sharply at day 3 (32.20%) and then declined rapidly.

At the genus level (Fig. 1a), *Enterococcus* (14.15%), *Proteus* (12.07%), and *Akkermansia* (11.11%) were the most abundant. The mucus-degrading *Akkermansia* peaked at day 3 and was nearly depleted by day 35, whereas *Enterococcus* showed strong mid-stage enrichment. Commensal genera *Lactobacillus* (6.43%) and *Limosilactobacillus* (5.36%) were abundant initially and declined over time.

Spearman correlation identified time-dependent genera (mean relative abundance > 1%, |ρ| > 0.5, *P* < 0.05; Fig. 1b). *Proteus* (ρ = 0.81), *Morganella* (ρ = 0.67), *Escherichia* (ρ = 0.67), and *Enterococcus* (ρ = 0.66) were positively correlated with time, whereas *Duncaniella* (ρ = –0.86), *Muribaculum* (–0.83), *Bacteroide*s (–0.76), *Lactobacillus* (–0.60), and *Akkermansia* (–0.52) were negatively correlated. These divergent temporal responses highlight the sensitivity of core bacterial taxa to decomposition progression.

### Effects of carcass decomposition on microbial diversity and community structure

**Fig 2:**
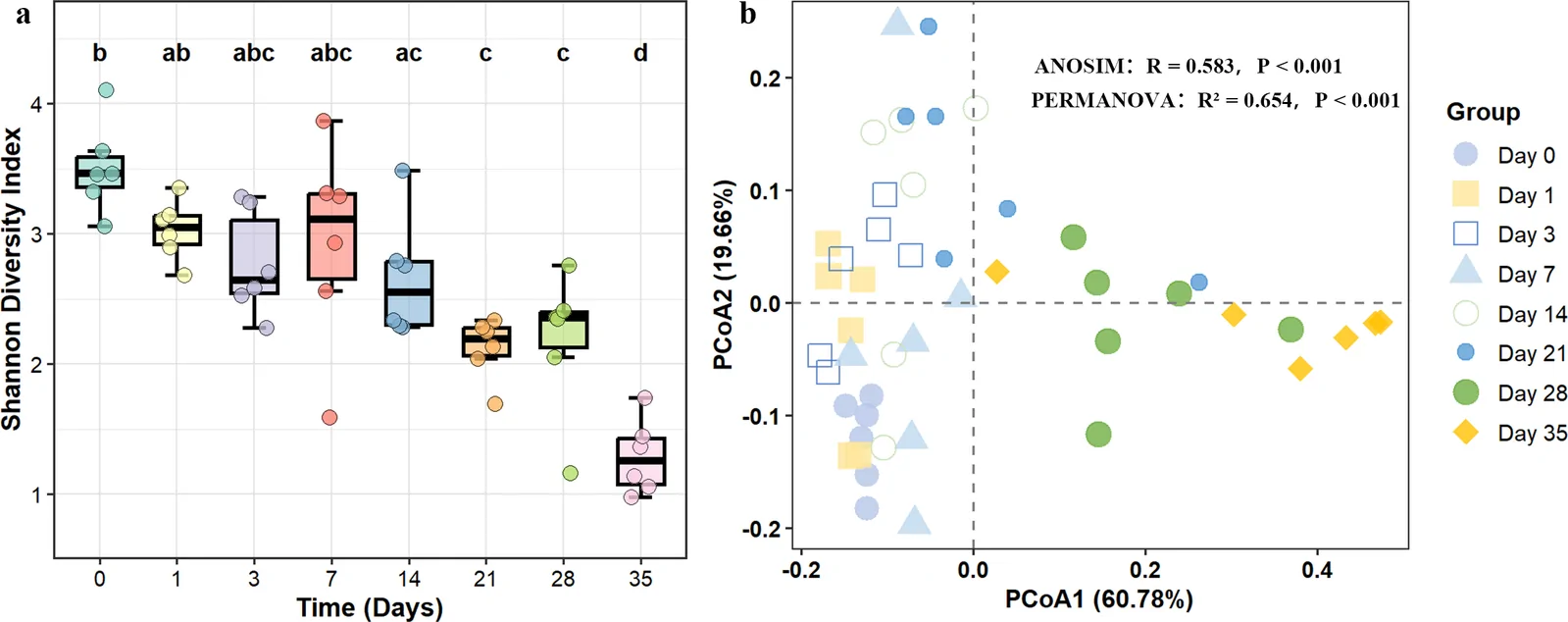
Temporal dynamics of bacterial diversity and community structure during decomposition. (**a**) Shannon diversity index across eight time points (days 0–35). Boxplots show mean ± SD (*n* = 6 per time point). One-way ANOVA: *F* = 13.86, *P* < 0.001. Different letters above boxes indicate significant differences (Tukey’s HSD, *P* < 0.05). (**b**) PCoA based on Bray-Curtis distances. Percentages in axes represent variance explained. Samples are colored by time point. Arrows indicate successional trajectory from early (days 0–3) to late (days 21–35) stages.

Bacterial Shannon diversity exhibited a continuous declining trend throughout carcass decomposition (Fig. 2a). The overall diversity decreased markedly from an initial value of 3.51 ± 0.35 at day 0 to the lowest level of 1.29 ± 0.29 at day 35. A sharp reduction occurred within the early decomposition stage (days 0–3), followed by moderate fluctuations in the middle phase and a further decline in the late stage. One-way ANOVA confirmed that decomposition time significantly altered bacterial Shannon diversity (*F* = 13.86, *P* < 0.001).

PCoA based on Bray-Curtis distance revealed clear temporal succession of bacterial community composition (Fig. 2b). The first two axes explained 60.78% and 19.66% of the total community variation, respectively. Samples were well clustered according to decomposition stages, with early (days 0–3), middle (days 7–14), and late (days 21–35) groups distinctly separated along the primary coordinate. PERMANOVA (*R*² =0.654, *P* < 0.001) and ANOSIM (*R* = 0.583, *P* < 0.001) further verified that decomposition time acted as a dominant driver shaping bacterial community assembly and causing significant temporal differentiation.

### Temporal dynamics and succession patterns of antibiotic resistome

**Fig 3:**
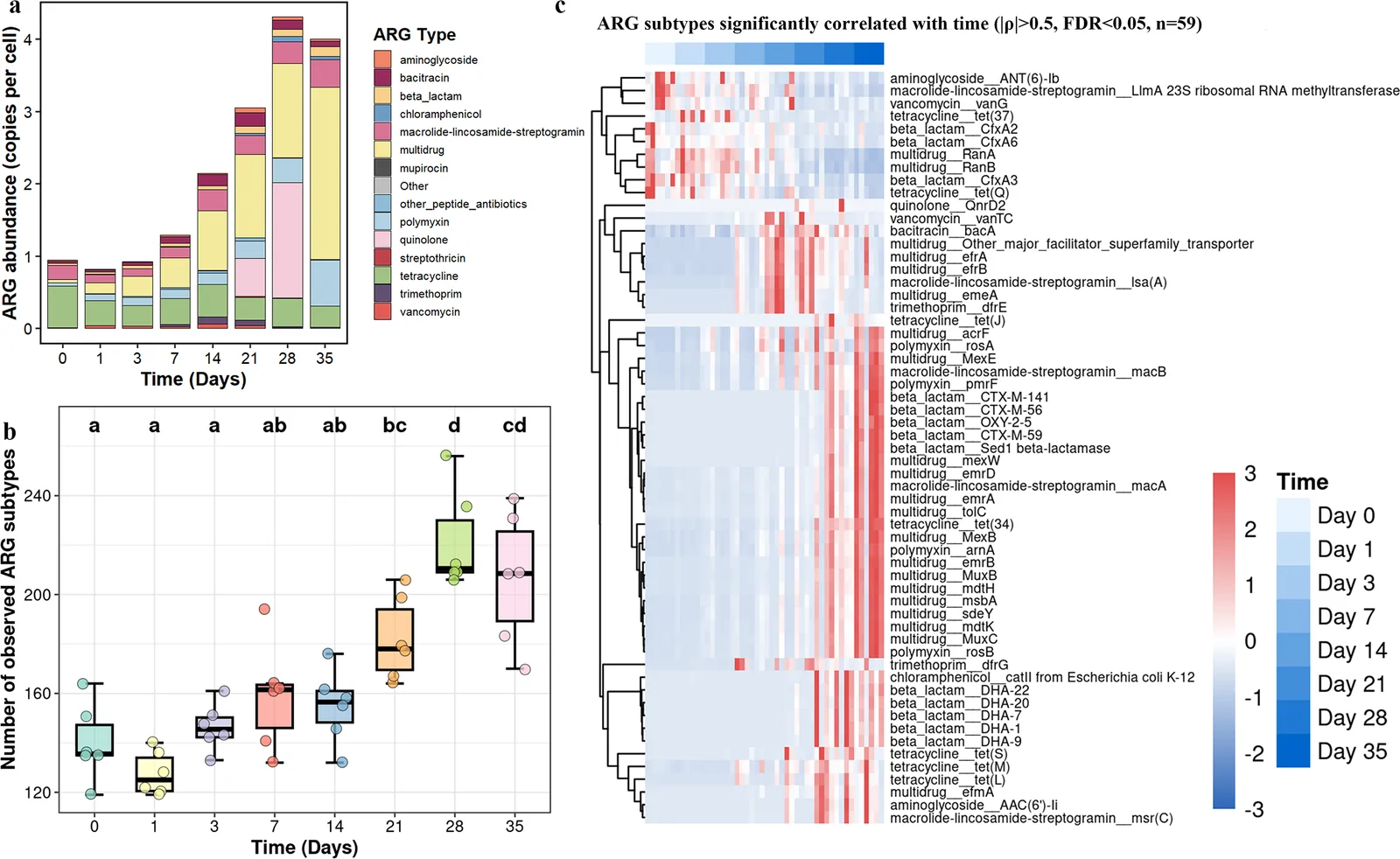
Temporal dynamics of antibiotic resistance genes during decomposition. (**a**) Stacked bar plot of relative abundance of ARG types across eight time points (days 0–35). Only types with mean abundance >1% are shown individually; others are grouped as “Other.” (**b**) Boxplot of observed ARG subtypes per sample. Boxes represent mean ± SD (*n* = 6 per time point). Different letters above boxes indicate significant differences (one-way ANOVA followed by Tukey’s HSD, *P* < 0.05). (**c**) Heatmap of 59 ARG subtypes significantly correlated with decomposition time (Spearman |ρ| > 0.5, *P* < 0.05).

A total of 24 ARG types and 616 ARG subtypes were detected. The dominant types (in descending order) were multidrug, tetracycline, quinolone, MLS, polymyxin, bacitracin, beta-lactam, trimethoprim, vancomycin, and aminoglycoside (Fig. 3a). Multidrug, polymyxin, and beta-lactam increased continuously; MLS declined slightly at day 3 and then increased; quinolone remained low before day 14, surged to a peak at day 28, and then rapidly declined; tetracycline started high, decreased at day 3, fluctuated, and then declined again; bacitracin and trimethoprim peaked at day 21; vancomycin and aminoglycoside remained low. Nine of the 12 predominant ARG types were significantly positively correlated with time (FDR < 0.05; Fig. S3).

Hierarchical clustering of the top 150 abundant ARG subtypes produced six stage-specific clusters (Fig. S4). Initial stage (days 0 and 1): tetracycline and MLS subtypes dominated. Early stage (days 3–7): multidrug-related subtypes and functional resistance genes [bacA and lsa(A)] were enriched. Middle stage (days 14–21): multidrug efflux pumps accumulated; quinolone resistant gene QnrD1 increased from day 21 to day 28. Late stage (days 28–35): a high-abundance module of QnrD1, emrB/emrA, and rosA/rosB emerged.

**Fig 4:**
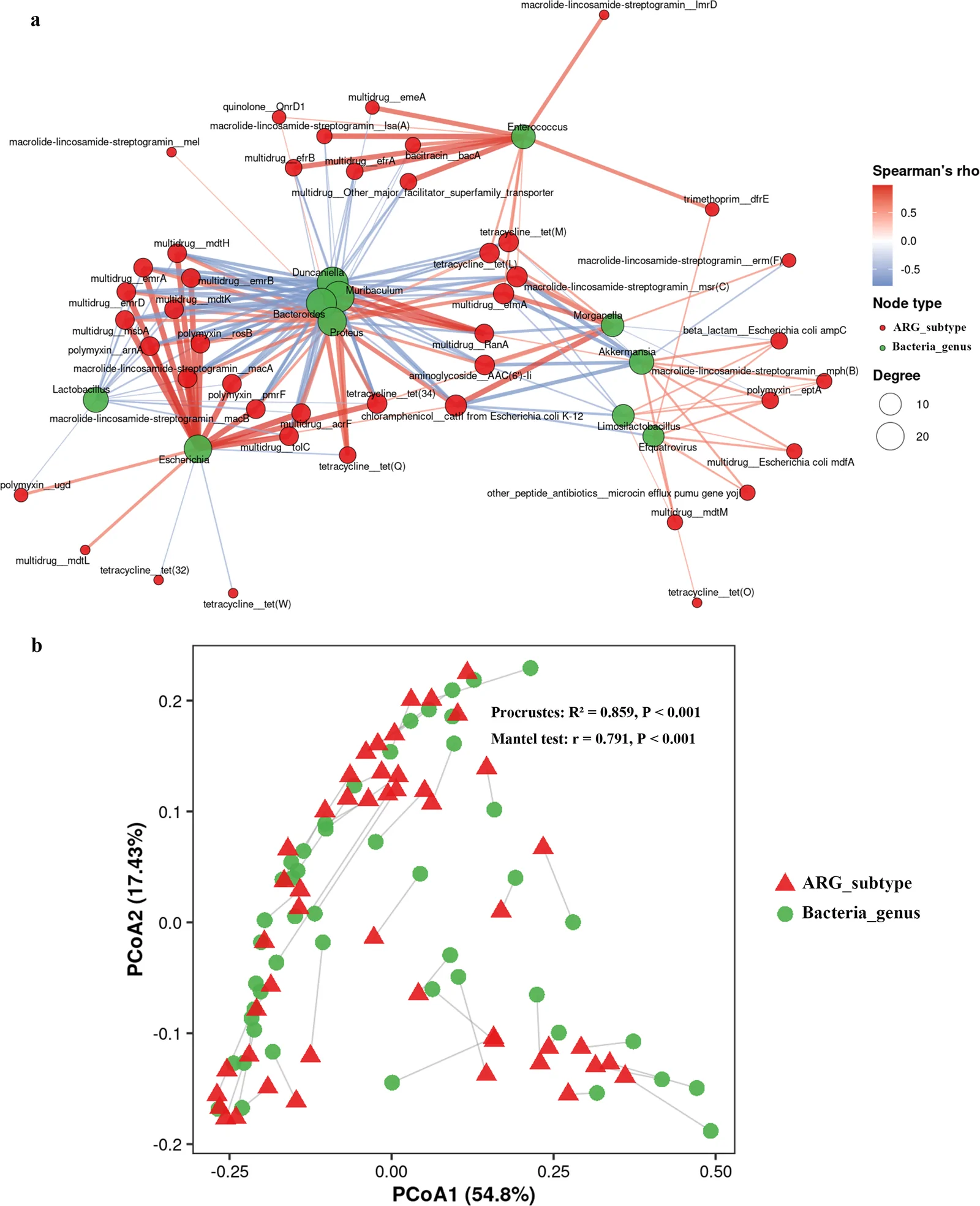
Co-occurrence network and Procrustes analysis of bacterial genera and ARG subtypes. (**a**) Spearman correlation network (|ρ| > 0.5, FDR < 0.05). Initially, 12 bacterial genera (mean relative abundance > 1%) and 50 ARG subtypes (highest total copy number) were considered. After removing isolated nodes without significant edges, the final network contains 11 bacterial genera (green) and 44 ARG subtypes (red), with 195 edges (87 positive, 108 negative). Edge color: red = positive, blue = negative; edge width proportional to |ρ|. Node size is proportional to degree. (**b**) Procrustes analysis of bacterial community (green) vs ARG subtype composition (red) based on Bray-Curtis distances. Lines connect matched samples. The high concordance is indicated by Procrustes *R*² =0.859 (*P* < 0.001) and Mantel test *r* = 0.791 (*P* < 0.001).

ARG subtype richness changed significantly over time (ANOVA: *F*  =  21.05, *P*  <  0.001). Richness decreased from day 0 to day 1, then increased steadily, peaked at day 28, and declined slightly by day 35. Tukey’s HSD confirmed that richness in middle and late stages was significantly higher than in the early stage (*P*  <  0.05). Spearman analysis identified 59 time-sensitive ARG subtypes (|ρ|  >  0.5, *P*  <  0.05); 49 were positively correlated with time (e.g., multidrug__*MexB*, ρ  =  0.87) and 10 negatively (e.g., beta_lactam__*CfxA3*, ρ = –0.80). Their temporal distribution is shown in Fig. 3c, and detailed information is in Table S1.

### Co-occurrence network between bacterial genera and ARG subtypes

To characterize the relationships between dominant bacterial taxa and antibiotic resistance genes, we constructed a co-occurrence network using 12 bacterial genera (mean relative abundance >1%, unassigned taxa excluded) and the top 50 ARG subtypes with high copy numbers. After filtering significant Spearman correlations (|ρ| > 0.5, FDR < 0.05) and removing isolated nodes, the final network contained 55 nodes (11 bacterial genera and 44 ARG subtypes) and 195 edges, including 87 positive and 108 negative correlations. Correlation coefficients ranged from –0.794 to 0.984, and node degrees varied between 1 and 28 (Fig. 4a).

*Duncaniella* (degree = 28), *Muribaculum* (25), *Bacteroides* (24), *Proteus* (20), and *Escherichia* (18) were the bacterial genera with the highest network connectivity. Among ARG subtypes, chloramphenicol__*catII* (degree = 9), macrolide-lincosamide-streptogramin__*msr(C)* (8) and multidrug__*efmA* (8) showed prominent connections, followed by multiple multidrug and tetracycline resistance subtypes [e.g., *tet(M*), *tet(34)*, degree = 7]. *Escherichia* exhibited strong positive correlations with efflux pump genes (multidrug__*acrF,* ρ = 0.98; multidrug__*msbA*, ρ = 0.96) and polymyxin resistance genes (*arnA*, *pmrF*). By contrast, *Duncaniella* and *Muribaculum* were mainly negatively correlated with ARG subtypes. These distinct patterns indicate that bacterial succession modulates the dynamics of specific ARG subtypes.

### Concordance between bacterial community and resistome succession

Procrustes analysis and Mantel test based on Bray-Curtis distances were applied to quantify the coupling between bacterial community and ARG composition (Fig. 4b). Procrustes analysis indicated high consistency in their successional trajectories (*R*² = 0.859, *P* < 0.001). The Mantel test further confirmed a strong correlation (*r* = 0.791, *P* < 0.001). These findings demonstrate that shifts in bacterial community structure are accompanied by coordinated changes in the resistome, verifying a robust co-successional pattern between bacteria and ARGs throughout carcass decomposition.

### Potential mechanisms driving ARG dynamics: roles of MGEs and HGT

**Fig 5:**
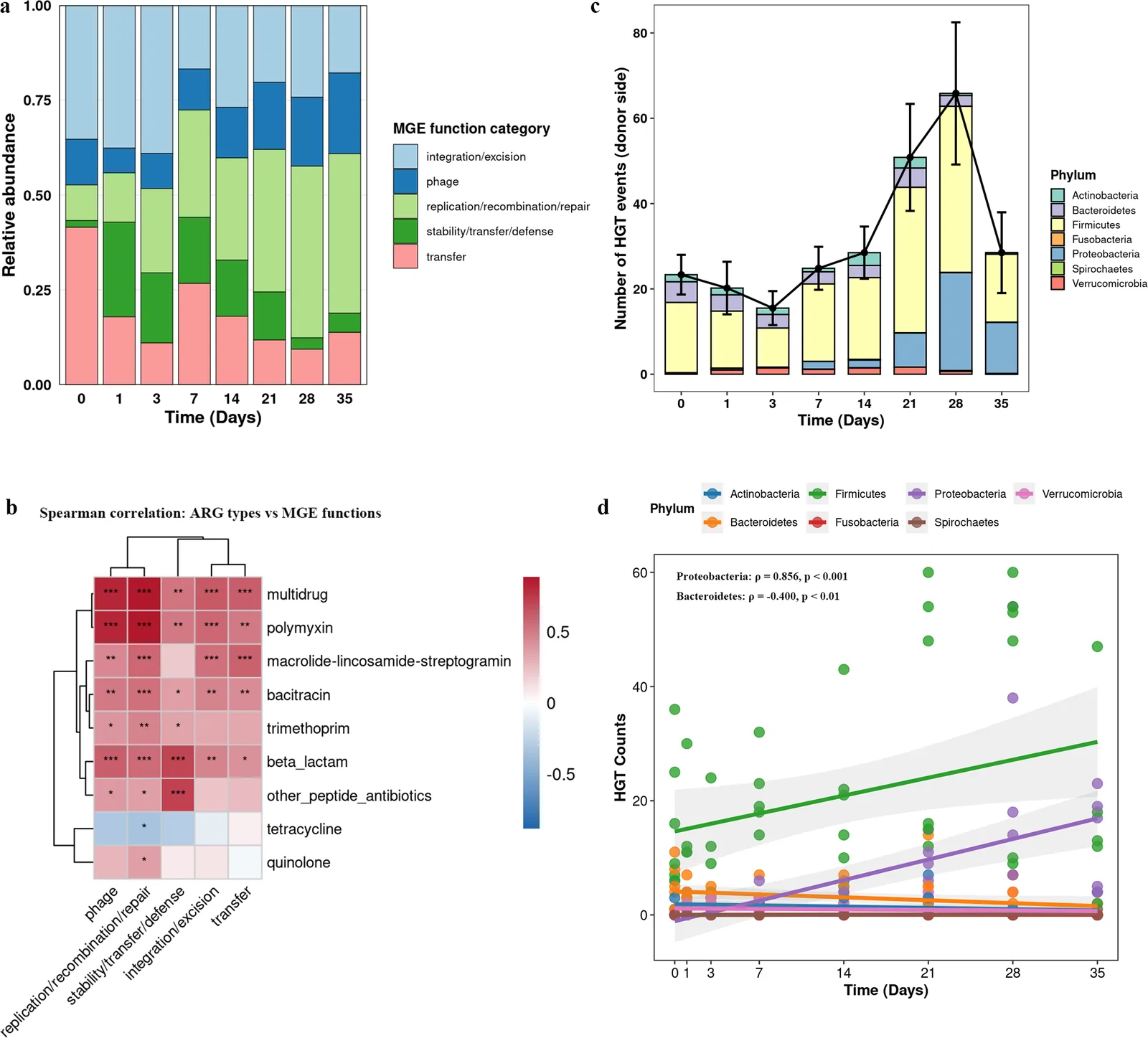
MGE functional dynamics, HGT donor phyla, and their correlations with ARGs. (**a**) Stacked area plot showing the temporal shifts in relative abundance of MGE functional categories (integration/excision, phage, replication/recombination/repair, stability/transfer/defense, transfer) across decomposition time points (days 0, 1, 3, 7, 14, 21, 28, and 35). (**b**) Heatmap of Spearman correlations between MGE functional categories and ARG types. Only significant correlations (FDR  <  0.05) are shown. Color intensity indicates the correlation coefficient (ρ). (**c**) Temporal dynamics of HGT events contributed by major bacterial phyla (Actinobacteria, Bacteroidetes, Firmicutes, Fusobacteria, Proteobacteria, Spirochaetes, and Verrucomicrobia). The total number of HGT events is shown in a bar plot, and the contributions of each phylum are stacked. (**d**) Spearman correlation coefficients (ρ) between decomposition time and HGT event counts for each donor phylum, with FDR-adjusted *P* values. Proteobacteria showed a strong positive correlation (ρ  =  0.856, *P*  <  0.001), whereas Bacteroidetes exhibited a negative correlation (ρ = –0.400, *P*  <  0.05).

The abundance of ARG-carrying MGEs increased during decomposition, with a temporary decrease on day 28 and a peak on day 35 (Fig. S5a). The proportion of MGEs involved in replication, recombination, and repair rose from 5.39% to 45.54% by day 28 and dominated in the mid-late stage. Phage-related MGEs reached 23.79% on day 35, whereas the initially dominant transfer function (48.36% on day 0) dropped sharply to 13.47% in the late stage (Fig. 5a).

Plasmids were the primary MGE carriers, while immedb and ICE persisted at low levels (Fig. S5b). Sankey analysis revealed covariation between MGE functions and carriers (Fig. S5d). MGE richness peaked on day 21, with significantly higher values on day 3 and day 21 than at the initial state (Fig. S5c). HGT frequency bottomed out on day 3 and peaked on day 28, matching the variation of ARG abundance (Fig. S6b). HGT donors shifted from Firmicutes (75.82%, day 0) to Proteobacteria (57.68%, day 35) (Fig. S6a), and Proteobacteria-derived HGT events were strongly correlated with time (ρ = 0.862, *P* < 0.001; Fig. 5d).

Procrustes and Mantel tests verified synchronous succession between MGE and ARG communities (*M*² = 0.111, *r* = 0.889, *P* < 0.001; Fig. S7a). PCoA showed the two communities clustered in the early stage and diverged in the late stage. Their compositional variation was strongly correlated (*r* = 0.747, *P* < 0.001; Fig. S7b). Plasmids, GPDs, and ICEs were all positively correlated with total ARG abundance (Fig. S5c).

Heatmaps showed distinct correlations between MGE functions and ARG types (Fig. 5b). Replication/recombination/repair MGEs correlated strongly with multidrug and β-lactam ARGs; phage-related MGEs were associated with MLS and polymyxin ARGs. MGEs with transfer and integration/excision functions had moderate positive links to multiple ARGs. Overall, MGE dynamics closely accompanied ARG succession, indicating MGE-mediated HGT promotes ARG accumulation in the mid-to-late decomposition stages.

## DISCUSSION

Microbial communities associated with buried carcass decomposition play a crucial role in nutrient cycling (48, 49). Although temporal succession studies of carcass decomposition based on animal models have been widely conducted, most have focused on dynamic changes in community structure (50). Notably, intestinal feces serve as a core reservoir of endogenous ARGs and resistant microorganisms (15, 51), representing a key vector for resistome release and dissemination during decomposition. Nevertheless, systematic analyses of the co-succession patterns of bacterial communities and ARGs in this specific niche under burial conditions remain limited (52). The present study found that carcass decomposition not only drives directional succession of bacterial communities but may also promote resistome enrichment through HGT mediated by MGEs. These findings provide a basis for understanding the microecological and resistome evolution mechanisms during buried carcass decomposition, further enrich the theoretical understanding of resistance gene dissemination under natural disturbance, and offer a reference for assessing the ecological effects of carcass burial.

### Bacterial community succession during carcass decomposition in buried environments

During carcass decomposition, the α-diversity of the microbial community decreased overall, with significantly lower values in the later stage than in the early stage, reflecting enhanced environmental filtering and niche occupation by dominant taxa. PCoA revealed clear separation of β-diversity among decomposition stages, with community structure shifting regularly over time. This successional pattern is highly consistent with previous studies (53), indicating that the shifting availability of nutritional substrates throughout decomposition drives stage-specific restructuring of the intestinal microbial community.

Carcass decomposition markedly altered bacterial community structure, with Firmicutes, Proteobacteria, and Bacteroidetes dominating throughout the process, consistent with previous reports (54). Firmicutes and Bacteroidetes, dominant in the early stage, are major gut phyla capable of degrading macromolecular organic matter (55, 56); after host death, intestinal bacteria immediately began to participate in decomposition (5). Proteobacteria increased continuously from an initial 8.25% to 76.20% by day 35, becoming the dominant group in the late stage. Proteobacteria are mostly facultative anaerobic, copiotrophic microorganisms that adapt well to the hypoxic environment and organic matter enrichment associated with decomposition. In contrast, Bacteroidetes dropped from 28.50% to 1.05%, and Actinobacteria also declined. Bacteroidetes depend on the neutral, mildly anaerobic gut environment and polysaccharide substrates (55). After death, intestinal barrier disruption, pH/redox changes, and toxic metabolites (e.g., ammonia, putrescine) destroy their survival niche; the late-stage substrate shift from polysaccharides to protein-based humic substances, combined with competitive suppression by stress-tolerant saprophytes (e.g., Proteobacteria), likely drives their severe decline. The decrease in Actinobacteria is likely related to their oligotrophic adaptation, which may be unsuited to the nutrient-rich environment of decomposition (57).

At the genus level, early-stage anaerobes such as *Enterococcus*, *Akkermansia*, and *Lactobacillus* declined over time, while Proteobacteria members (*Proteus*, *Morganella*, and *Escherichia*) became enriched in the middle-to-late stage. These facultative/obligate anaerobes adapt well to the closed, hypoxic burial system. Unlike open-system studies that observed late-stage aerobic enrichment, our focus on intestinal fecal environments, combined with oxygen consumption by ammonia accumulation, likely prolonged anaerobic dominance. Environmental heterogeneity can lead to markedly different decomposer community structures (54). Compared with indoor constant-temperature sterile simulation studies (50), core intestinal genera were largely consistent, yet the relative abundances and successional trends of dominant taxa differed. This discrepancy may be attributed to multiple environmental filters in our field burial system, including soil physicochemical perturbations, oxygen infiltration, and natural temperature/humidity fluctuations, which highlight burial-specific selective pressures.

Previous studies have shown that uniform nutrient disturbance and homogenized microenvironments from carcass input can drive convergent succession across different backgrounds (58). Shared nutrient substrates and physicochemical stresses during decomposition act as core drivers of this pattern. Notably, the core successional taxa identified here were strongly linearly correlated with decomposition time, suggesting their potential as universal microbial indicators for PMI estimation.

### Temporal succession and diversity dynamics of the resistome

During carcass decomposition, ARG composition exhibited a highly predictable successional pattern. Multidrug, tetracycline, quinolone, and MLS were the dominant ARG types, consistent with previous reports (18, 24). Multidrug, beta_lactam, and polymyxin ARGs continuously increased, while tetracycline ARGs gradually decreased from an initially high abundance. The high abundance of tetracycline resistance genes in the early stage may reflect the intrinsic features of the gut resistome in farmed animals, a pattern likely linked to the use of feed additives or therapeutic antibiotics in animal husbandry (15). Quinolone ARGs exhibited a peak in the middle-to-late stage. These dynamic trends partially align with previous studies (18), and the stage-specific succession pattern reflects directional selection driven jointly by substrate supply, oxygen conditions, and community composition (59). We observed in a burial environment a continuous decline of tetracycline ARGs and a short-term outbreak of quinolone ARGs, which may be related to the more persistent anoxic environment and ammonia accumulation under burial conditions.

Analysis of time-sensitive ARG subtypes further confirmed the directionality of enrichment. Among 59 time-sensitive subtypes, 49 were positively correlated with decomposition time, especially the sustained enrichment of multidrug efflux pump genes (e.g., *MexB*) and beta_lactamase genes (e.g., *Sed1*), indicating that the decomposition process selectively enriches specific resistance genes. Subtype succession exhibited clear phasing: initial dominance by gut-inherent resistance genes [e.g., *tet(Q)*, *tet(W)*, and *erm(F)*], likely originating from the release of intestinal native flora after death; a middle stage characterized by continuous accumulation of multidrug efflux pump genes; and a late stage forming a high-abundance module centered on *QnrD1*, *emrAB,* and *rosAB*. This fine-scale subtype succession order provides potential biomarkers for inferring decomposition stages using the resistome.

### Co-occurrence network of bacterial genera and ARG subtypes, and concordance between bacterial community and resistome succession

Network analysis indicated that microbial succession is a core biological factor regulating resistome structure. *Escherichia* and *Proteus* (Proteobacteria) showed strong positive correlations with multidrug efflux pump genes (*acrF* and *msbA*) and polymyxin resistance genes (*arnA* and *pmrF*), consistent with the notion that Proteobacteria are main ARG hosts (18) and may suggest *Escherichia* as a hub for multidrug resistance dissemination. In contrast, native anaerobes (*Duncaniella* and *Muribaculum*) were negatively correlated with ARGs, implying that succession both enriches resistant taxa and eliminates sensitive ones. Bacteroides’ negative correlations with various ARGs suggest that the original resistance reservoir may be disrupted, not passively released, under extreme decomposition disturbance. The coexistence of positive and negative associations indicates resistome dynamics are shaped by succession, niche competition, and gene transfer (59). Highly connected ARGs [e.g., *catII*, *msr(C)*, *efmA*, *tet(M)*] are broad-spectrum transmissible markers and may serve as marker genes.

Bacterial communities are major drivers of ARG distribution and serve as potential hosts (60–62). Procrustes analysis (*R*² = 0.859) and Mantel test (*r* = 0.791) revealed a strong co-successional relationship between bacterial communities and the resistome, suggesting community turnover drives resistome dynamics. This coupling strength is consistent with soil/water decomposition studies (18, 24, 59), and higher than that of Yu et al. (59), possibly due to our focus on intestinal feces as the core ARG reservoir. Gut microbiota are important environmental ARG hosts (63). Early stage (Firmicutes/Bacteroidetes dominance) coincided with tetracycline/MLS enrichment; middle-to-late stage (expansion of *Proteus*, *Escherichia*, *Morganella*) accompanied accumulation of multidrug, polymyxin, and efflux pump ARGs. This coordinated succession indicates that microbial community turnover is a core driver of resistome dynamics, with environmental filtering reinforcing the synergy (14, 53). This is consistent with Forsberg et al. (64), who proposed that bacterial community composition is a major determinant of resistome content.

### Potential mechanisms of resistome enrichment mediated by MGEs and HGT

During carcass decomposition, the enrichment of the resistome may be an active process driven by MGE-mediated HGT rather than passive release. MGE abundance, functional composition, and HGT frequency changed synchronously in the middle-to-late stage, coinciding with the ARG peak. In the early stage, MGEs were dominated by “transfer” functions (integrases, conjugation proteins), consistent with their release from lysed gut bacteria. In the middle-to-late stage, “replication/recombination/repair” and “phage” functions became enhanced. Replication and recombination are prerequisites for plasmid and transposon amplification (65, 66), while enhanced phage activity may accelerate cross-strain ARG transmission via transduction (23, 67). The specific association of phage-related MGEs with MLS and polymyxin ARGs suggests that different MGE modules may mediate particular resistance classes. HGT frequency decreased to a minimum on day 3, then increased continuously to a peak on day 28, coinciding with the highest ARG abundance and richness. This window matched the shift of dominant bacteria from Firmicutes to Proteobacteria. Proteobacteria-derived HGT events increased significantly with time, suggesting that Proteobacteria may be the predominant HGT donors in the middle-to-late stage. Given their high potential for horizontal gene transfer (68) and massive proliferation during decomposition, Proteobacteria likely contributed substantially to the stage-specific resistome expansion.

Key MGE carriers (plasmids, GPDs, and ICEs) all showed significant positive correlations with ARG abundance. Plasmids, as classical conjugative vectors, may have continuously participated in ARG spread (65, 69); ICEs, with their “integration-excision-transfer” life cycle, may promote cross-taxon dissemination from Firmicutes to Proteobacteria (70, 71); and the late-stage enrichment of phage-like GPDs partly explains the strong association of phage functions with MLS ARGs (72). Procrustes and Mantel tests revealed a significant co-succession between MGEs and ARGs. Functional resistance analysis further indicated that replication/recombination functions are linked mainly to multidrug and β-lactam ARGs, while phage functions are associated with MLS and polymyxin ARGs. This targeted matching suggests that specific MGE modules activated in the middle-to-late stage may directionally promote horizontal diffusion of corresponding resistance genes. Moreover, at day 35, ARG abundance and diversity remained higher than initial levels, suggesting that carcass-derived resistomes may persist in soil environments. Carcass decomposition drastically alters pH and ammonia, creating environmental filtration pressure that favors ammonia-tolerant, high-pH-tolerant Proteobacteria. This deterministic assembly pattern is consistent with previous findings (18, 59, 73) and provides a plausible explanation for the directional enrichment of resistance genes.

### Limitations and perspectives

This study uncovered a correlated ecological cascade encompassing microbial community succession, activation of MGEs and HGT events, and subsequent resistome enrichment throughout buried carcass decomposition, providing supplementary theoretical insights into resistome dynamics shaped by natural carcass decomposition disturbance. Nevertheless, several limitations remain in this study. First, the temporal correlation analysis based on metagenomic sequencing can only demonstrate the coupling relationships among bacterial communities, MGEs, and ARGs but cannot confirm the actual transfer efficiency and causal linkages between specific MGE-ARG pairs. Further functional verification experiments are therefore required to validate these observed associations. Second, this study focused on the endogenous evolution of intestinal fecal resistomes during decomposition, while cross-interface resistome dissemination via carcass leachate and soil migration was not systematically explored. This leaves the full field-scale transmission chain of carcass-associated resistomes uncharacterized. Third, the current investigation covered only a 35-day short-term decomposition period; the long-term persistence of enriched ARGs and their subsequent ecological impacts on soil microbial communities remain unclear. Future studies should combine pure culture, *in situ* tracing, and functional validation to confirm HGT mechanisms, and extend long-term, multi-interface sampling to quantify environmental diffusion and persistence of resistomes from buried carcasses. Overall, this study enhances understanding of microbial-resistome co-succession under burial disturbance and provides a theoretical reference for assessing the ecological consequences of natural disturbance-driven resistome enrichment during carcass decomposition.

### Conclusion

In this study, using a field-buried rat model, we systematically analyzed the co-succession patterns and potential driving mechanisms of intestinal bacterial communities and the antibiotic resistome during carcass decomposition. The bacterial community exhibited a directional succession from native anaerobes to facultative anaerobes such as Proteobacteria, with species diversity and community structure showing stage-specific characteristics. The resistome displayed clear temporal differentiation: tetracycline and MLS ARGs dominated the early stage, while multidrug, β-lactam, and polymyxin resistance genes continuously accumulated in the middle-to-late stage, and quinolone resistance genes showed a transient enrichment peak. Procrustes analysis and the Mantel test confirmed a high degree of synchrony between bacterial community and resistome dynamics. Co-occurrence network analysis suggested that Proteobacteria, particularly *Escherichia*, may serve as important potential hosts of multidrug resistance genes. At the mechanistic level, MGE functions shifted from a “transfer” dominance in the early stage to “replication/recombination/repair” and “phage”-related functions in the middle-to-late stage, and the frequency of HGT events coincided with the peak of ARG abundance. Proteobacteria likely act as the core donors for the horizontal dissemination of resistance genes. Collectively, this study preliminarily identified a correlated sequential pattern consisting of community turnover, functional remodeling of MGEs, elevated HGT activity, and resistome enrichment within the burial microhabitat, which advances the theoretical basis for characterizing resistome succession shaped by natural decomposition disturbance in buried carcass ecosystems.

## Data Availability

The raw sequencing data generated in this study has been deposited in the NCBI Sequence Read Archive (SRA) under accession number PRJNA1293747. Additional supplemental data are posted with this article.

## SUPPLEMENTAL MATERIAL

Supplemental material msphere.00437-26-s0001.pdf Fig. S1–S7 and Table S1.

ASM does not own the copyrights to Supplemental Material that may be linked to, or accessed through, an article. The authors have granted ASM a non-exclusive, world-wide license to publish the Supplemental Material files. Please contact the corresponding author directly for reuse.
