# Supplementary material for: Temporal succession of microbiomes and resistomes during buried rat carcass decomposition
Source: mSphere. 2026 Aug 12;11(9):e00437-26. doi: 10.1128/msphere.00437-26 (PMC13621866; doi:10.1128/msphere.00437-26)
Supplement: Supplementary file 1 [file msphere.00437-26-s0001.pdf]

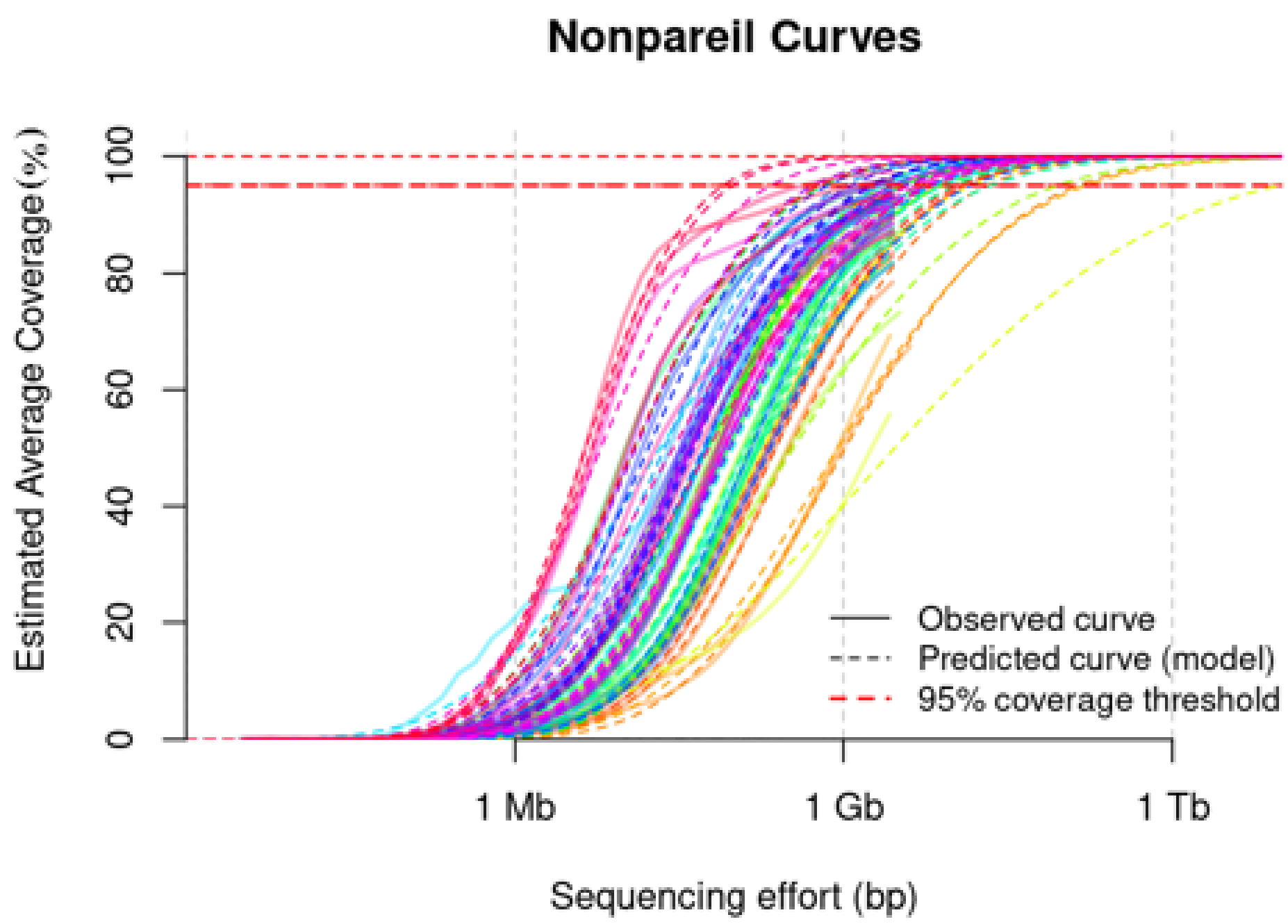

Fig. S1. Nonpareil curves of sequencing coverage saturation. Most samples reached near-plateau coverage, confirming that the sequencing depth was adequate for microbial diversity analysis.

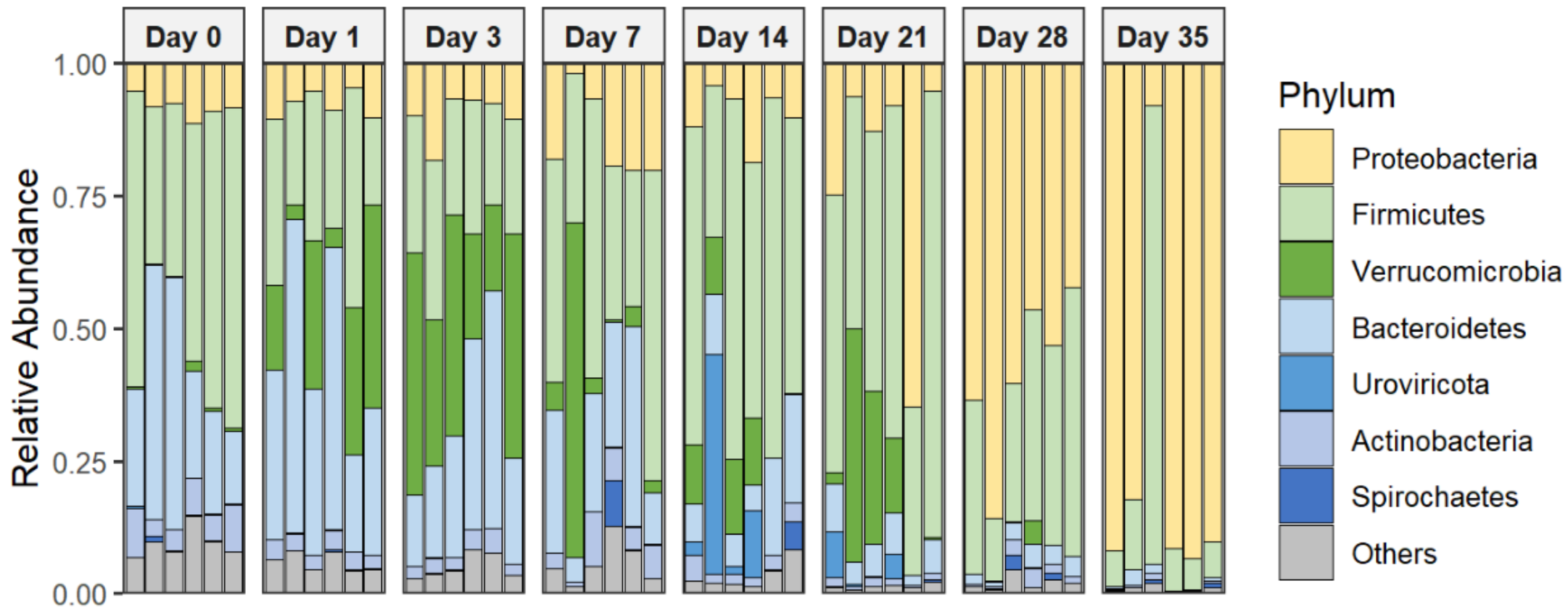

Fig. S2. Bacterial community composition at the phylum level during different stages of carcass decomposition. Only those taxa with mean relative abundance more than 1% are shown.

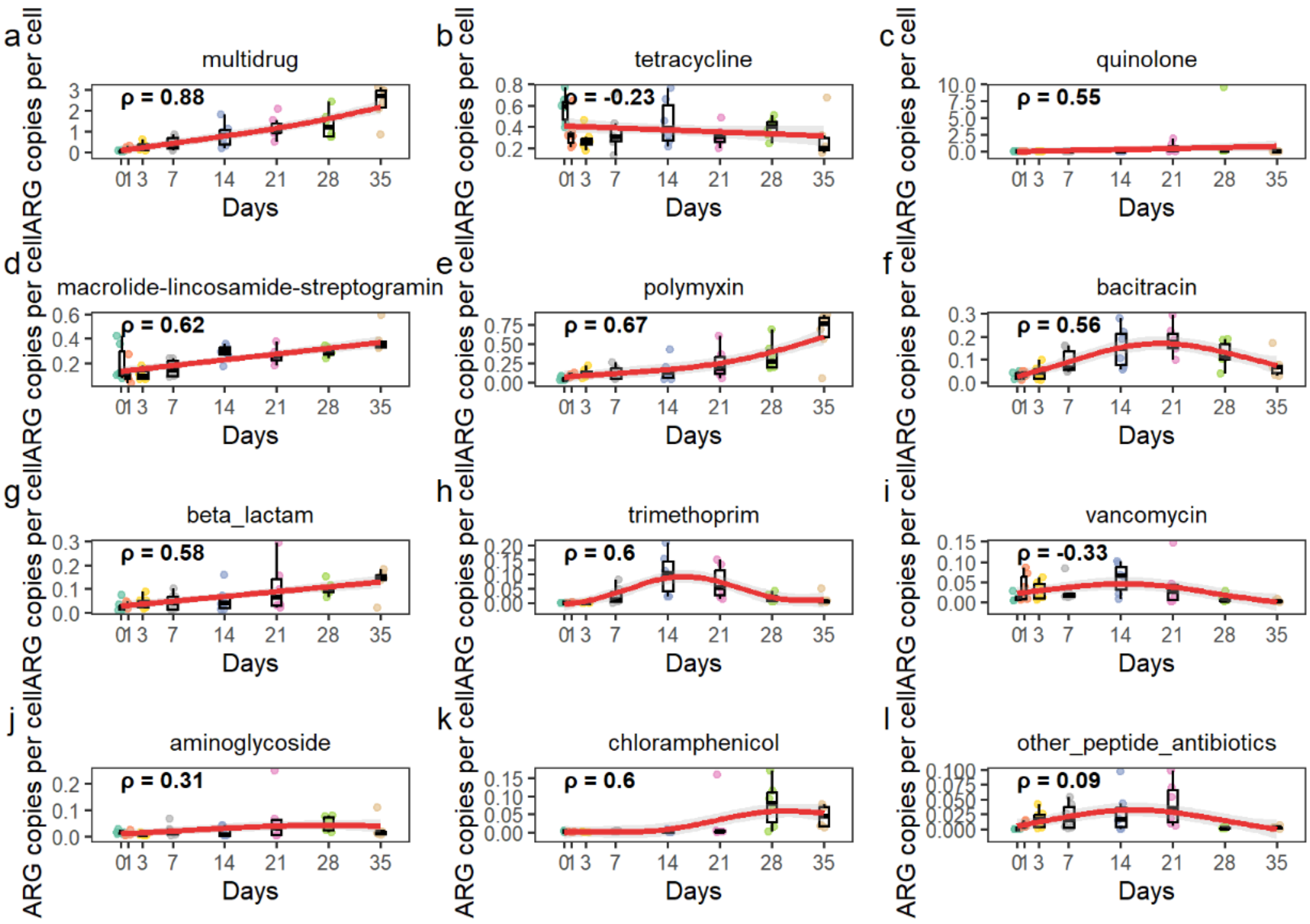

Fig. S3. Spearman correlation between the abundance of major ARG types and decomposition time. Only the 12 most abundant ARG types (occurrence rate ≥ 10%, ranked by total copy number) were analyzed. For each type, individual samples are shown as gray points, boxplots summarize the distribution at each time point (Day 0, 1, 3, 7, 14, 21, 28, 35), and the red line represents a smooth fit with a 95% confidence interval (shaded area). Spearman's rank correlation coefficient (ρ) and FDR adjusted P value are indicated at the top of each panel. Two types, other_peptide_antibiotics ($\rho = 0.09$, FDR > 0.05) and tetracycline ($\rho = -0.23$, FDR > 0.05), were not significantly correlated with decomposition time.

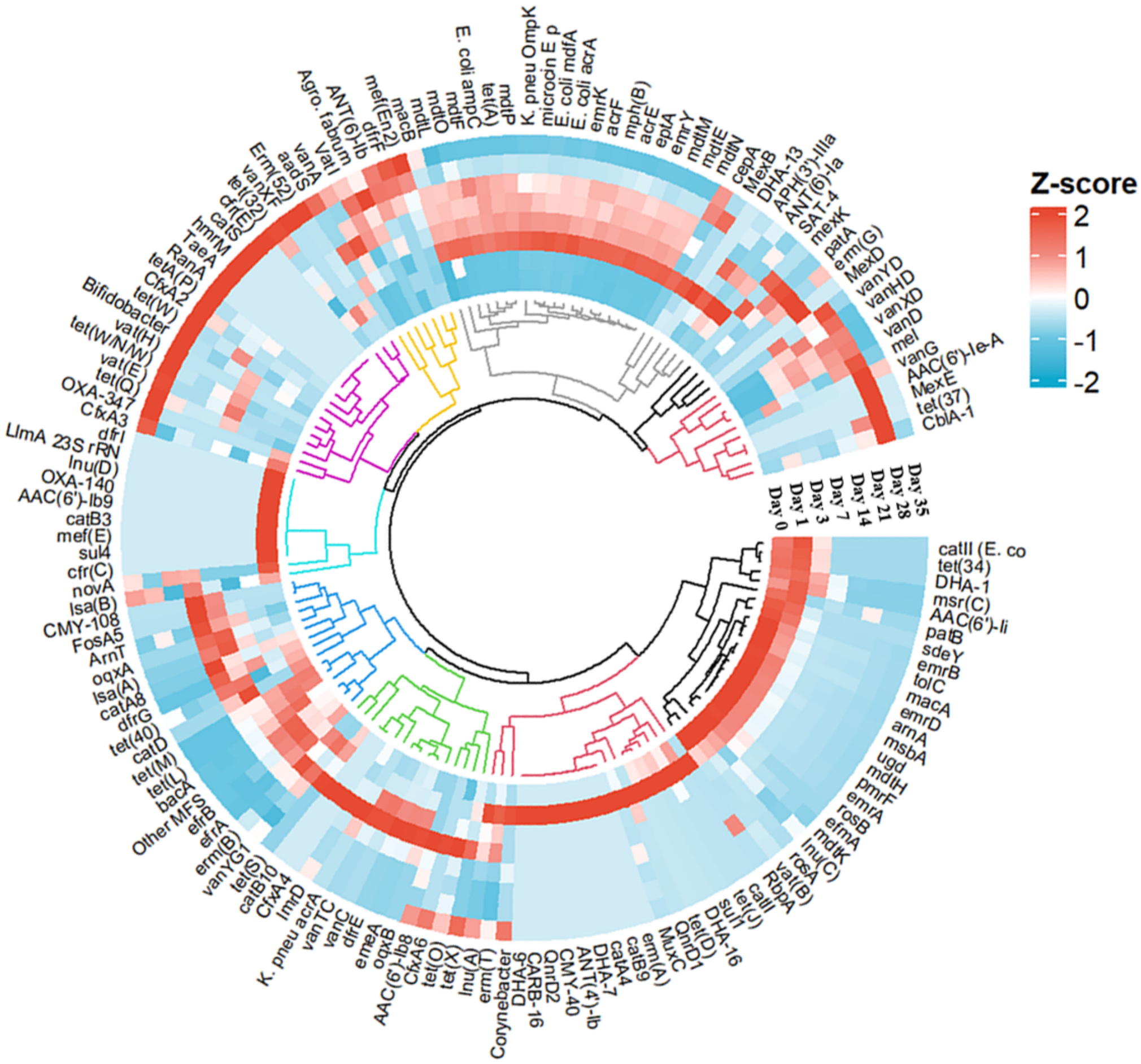

Fig. S4. Circular heatmap depicting abundance patterns of high-abundance ARG subtypes during carcass decomposition. In the plot, each colored segment represents the abundance level of an ARG subtype across the decomposition timeline, normalized as a Z-score (color gradient: red indicates high abundance, blue indicates low abundance). The outer ring labels individual ARG subtypes, while hierarchical clustering branches illustrate similarities in abundance patterns among these subtypes. This visualization reveals dynamic abundance trends and clustering characteristics of the most abundant ARGs throughout the decomposition process.

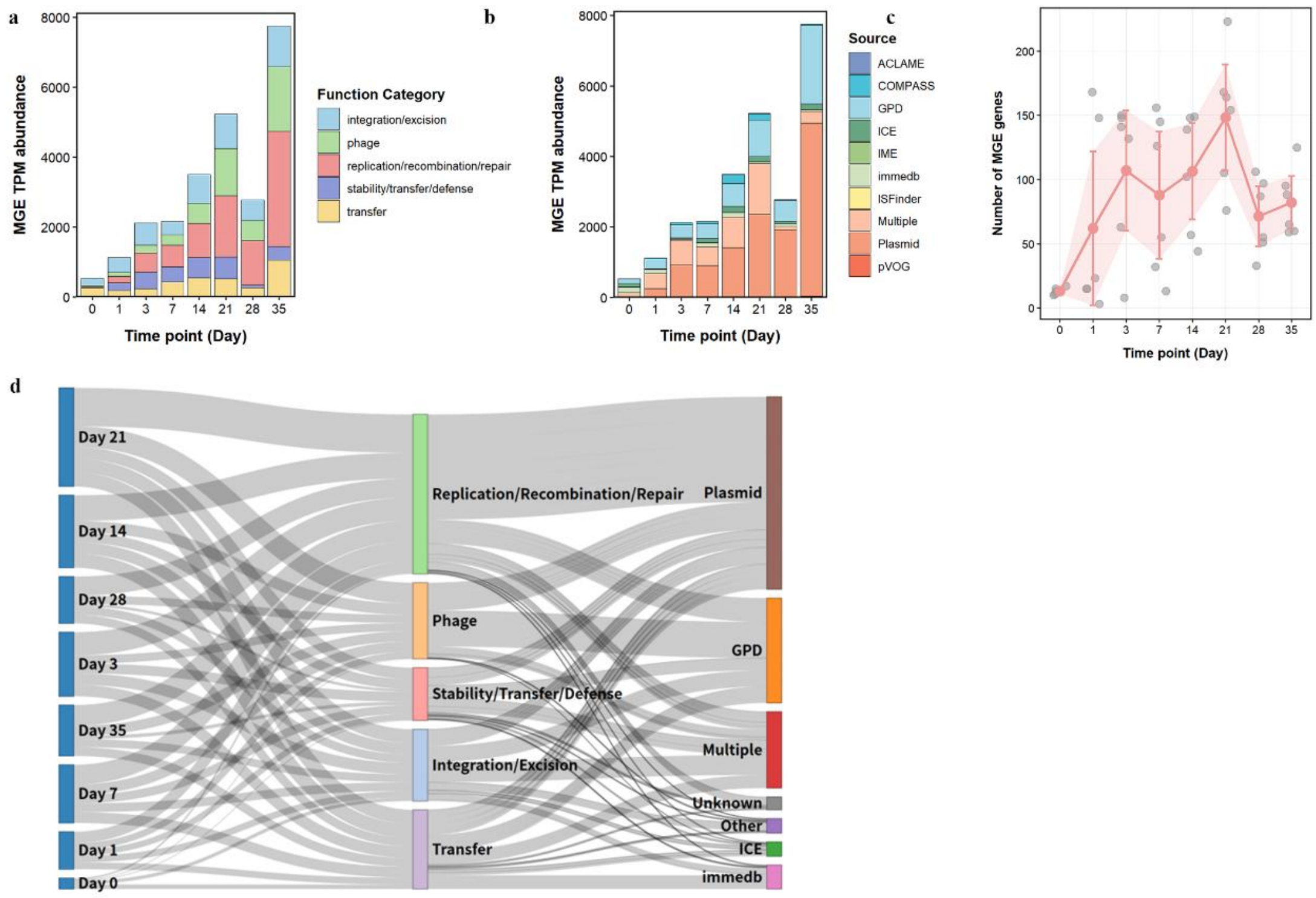

Fig. S5. Source composition, functional categories, and temporal dynamics of MGEs during carcass decomposition. (a) Temporal changes in the TPM abundance of MGEs categorized by functional class. (b) Stacked bar plot showing the relative abundance of MGEs by annotated source across different time points (Days 0–35). (c) Changes in the number of MGE genes over decomposition time. (d) Sankey diagram illustrating the association between MGE functional categories and their primary sources across the entire decomposition period.

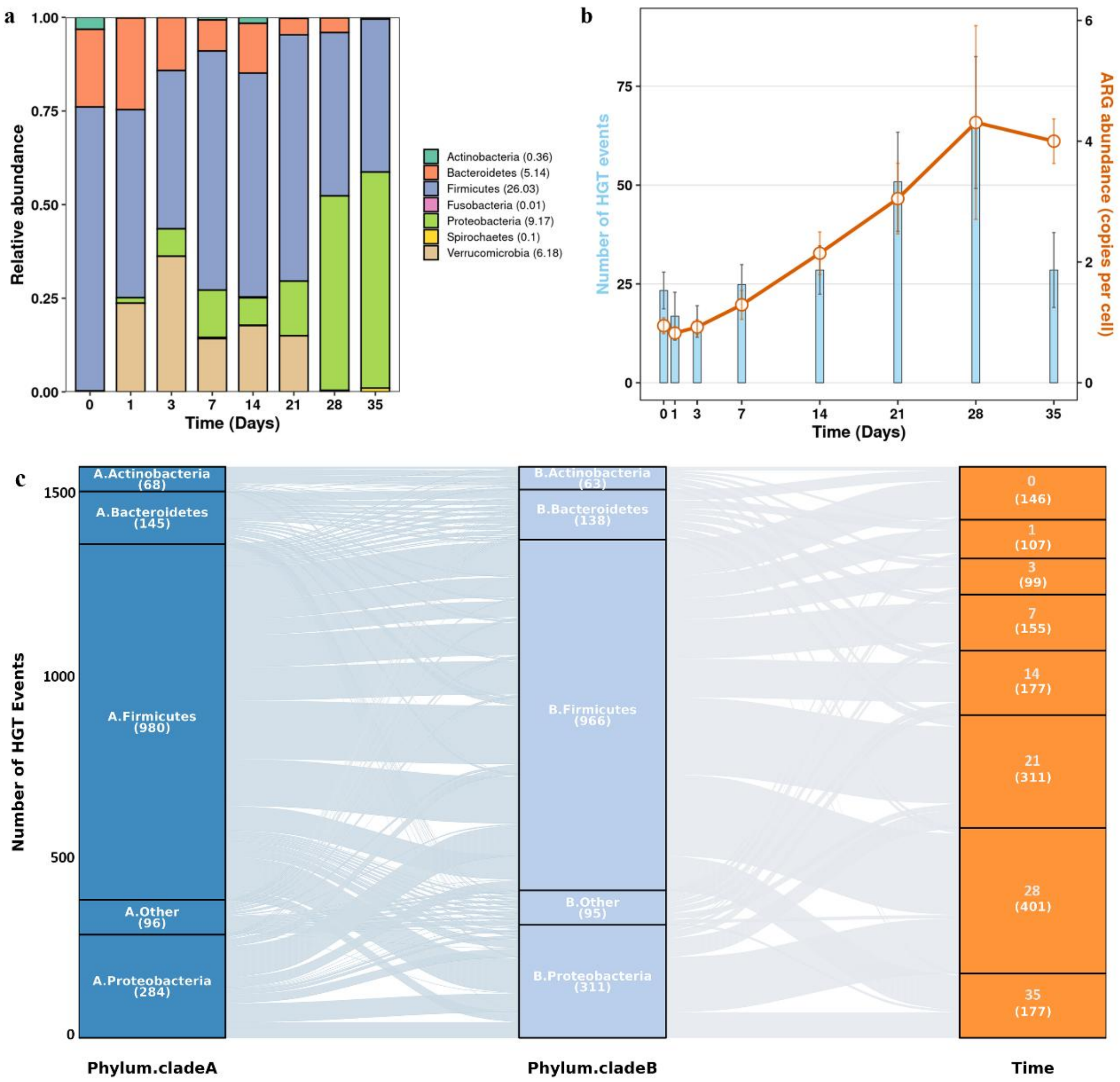

Fig. S6. Phylum-level composition and correlation characteristics of species involved in HGT events during carcass decomposition. (a) Temporal dynamics of the relative abundance of phyla involved in HGT events: A stacked bar plot illustrates time-series changes in the relative abundance of bacterial phyla participating in HGT events throughout the decomposition period (Days 0–35). (b) Temporal dynamics of HGT events and ARG abundance. Bar plot shows the number of HGT events (left Y-axis), and line graph with error bars represents the mean ARG abundance (copies per cell, right Y-axis) across eight time points (Days 0, 1, 3, 7, 14, 21, 28, 35). (c) Sankey flow diagram illustrating the distribution of HGT events across major phyla within Clade A and Clade B lineages at different time points. The number of HGT events for each taxon is indicated in parentheses.

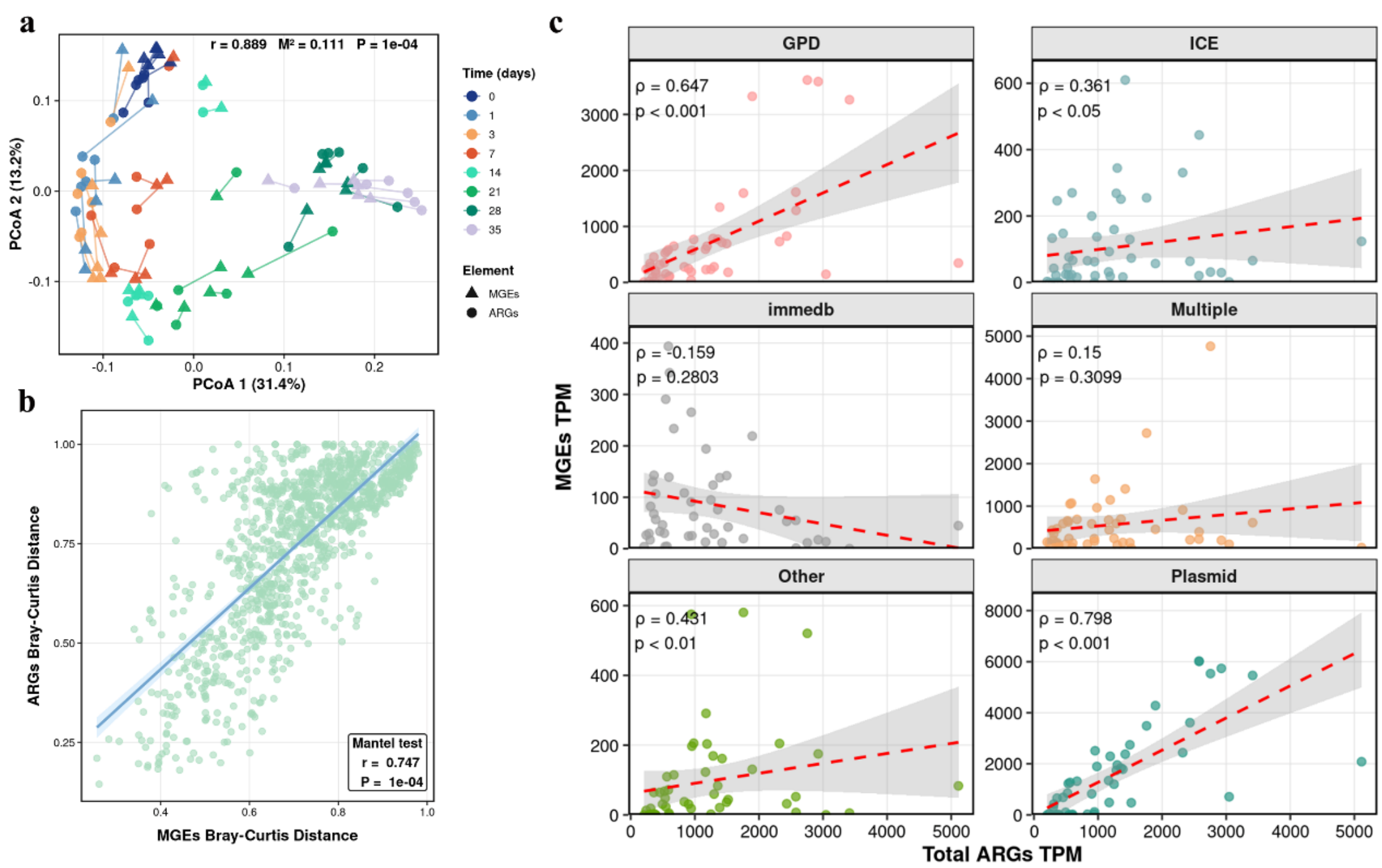

Fig. S7. Community-level associations and interactions between ARGs and MGEs. (a) Procrustes analysis: based on Bray–Curtis distance matrices, the PCoA plot illustrates the covariation of ARG and MGE community structures over time, where r denotes the correlation coefficient between the ordination axes and the original distance matrices, M^2 represents the goodness-of-fit of the Procrustes superimposition, and P indicates statistical significance. (b) Mantel test: this test quantifies the global correlation between the Bray–Curtis distance matrices of ARG and MGE communities (r = correlation strength; P = significance level), indicating significant covariation in their community structures. (c) Spearman correlation (ρ) and linear regression analysis show the relationship between the relative abundance of different MGE types (GPD, ICE, immedb, Multiple, Other, Plasmid) and total ARG abundance. The correlation coefficient (ρ) and significance (P) are provided for each association, highlighting co-abundance patterns between specific MGEs and ARGs.

Table S1 Spearman correlation coefficients (ρ) and significance levels for ARG subtypes that were significantly correlated with decomposition time (|ρ| > 0.5, FDR < 0.05). FDR adjustment was performed using the Benjamini-Hochberg method. Negative ρ values indicate decreasing abundance over time, while positive ρ values indicate increasing abundance.

ARG subtype	Spearman's ρ	P-value	FDR-adjusted P-value
aminoglycoside__ANT(6)-Ib	-0.620	2.58E-06	5.90E-06
bacitracin__bacA	0.558	3.79E-05	8.21E-05
beta_lactam__CfxA2	-0.540	7.41E-05	1.55E-04
beta_lactam__CfxA3	-0.796	1.38E-11	6.85E-11
beta_lactam__CfxA6	-0.704	2.33E-08	7.31E-08
chloramphenicol__catII from Escherichia coli K-12	0.689	6.31E-08	1.92E-07
macrolide-lincosamide-streptogramin__LlmA 23S ribosomal RNA methyltransferase	-0.646	6.92E-07	1.68E-06
macrolide-lincosamide-streptogramin__macB	0.761	3.34E-10	1.20E-09
multidrug__MexB	0.834	1.79E-13	2.93E-12
multidrug__MuxB	0.866	1.81E-15	1.14E-13
multidrug__MuxC	0.816	1.63E-12	1.21E-11
multidrug__Other_major_facilitator_superfamily_transporter	0.673	1.63E-07	4.61E-07
multidrug__RanA	-0.784	4.26E-11	1.69E-10

ARG subtype	Spearman's ρ	P-value	FDR-adjusted P-value
multidrug__RanB	-0.833	2.11E-13	2.93E-12
multidrug__acrF	0.680	1.07E-07	3.09E-07
multidrug__efrA	0.657	3.94E-07	1.02E-06
multidrug__efrB	0.665	2.55E-07	6.90E-07
multidrug__emrA	0.816	1.51E-12	1.20E-11
multidrug__emrB	0.832	2.30E-13	2.93E-12
multidrug__emrD	0.797	1.25E-11	6.49E-11
multidrug__mdtK	0.809	3.38E-12	2.01E-11
multidrug__mexW	0.780	6.29E-11	2.41E-10
multidrug__msbA	0.788	2.92E-11	1.24E-10
multidrug__sdeY	0.805	5.16E-12	2.92E-11
multidrug__tolC	0.813	2.19E-12	1.45E-11
polymyxin__arnA	0.785	4.01E-11	1.64E-10
polymyxin__pmrF	0.775	1.05E-10	3.91E-10
polymyxin__rosA	0.559	3.61E-05	7.95E-05
polymyxin__rosB	0.817	1.40E-12	1.19E-11
tetracycline__tet(37)	-0.555	4.17E-05	8.86E-05
tetracycline__tet(M)	0.645	7.67E-07	1.83E-06

ARG subtype	Spearman's ρ	P-value	FDR-adjusted P-value
tetracycline__tet(Q)	-0.648	6.46E-07	1.60E-06
vancomycin__vanG	-0.612	3.74E-06	8.39E-06
aminoglycoside__AAC(6')-Ii	0.752	7.23E-10	2.46E-09
beta_lactam__CTX-M-141	0.858	6.39E-15	2.54E-13
beta_lactam__CTX-M-56	0.838	1.15E-13	2.28E-12
beta_lactam__CTX-M-59	0.841	7.68E-14	2.24E-12
beta_lactam__DHA-1	0.827	4.61E-13	4.22E-12
beta_lactam__DHA-20	0.789	2.61E-11	1.15E-10
beta_lactam__DHA-22	0.814	1.99E-12	1.39E-11
beta_lactam__DHA-7	0.827	4.61E-13	4.22E-12
beta_lactam__DHA-9	0.756	5.17E-10	1.81E-09
beta_lactam__OXY-2-5	0.839	9.41E-14	2.24E-12
beta_lactam__Sed1 beta-lactamase	0.866	1.92E-15	1.14E-13
macrolide-lincosamide-streptogramin__lsa(A)	0.671	1.80E-07	4.98E-07
macrolide-lincosamide-streptogramin__macA	0.811	2.85E-12	1.79E-11
macrolide-lincosamide-streptogramin__msr(C)	0.830	3.11E-13	3.36E-12

ARG subtype	Spearman's ρ	P-value	FDR-adjusted P-value
multidrug__MexE	0.715	1.15E-08	3.70E-08
multidrug__efmA	0.802	7.11E-12	3.85E-11
multidrug__emeA	0.662	3.06E-07	8.09E-07
multidrug__mdtH	0.832	2.47E-13	2.93E-12
quinolone__QnrD2	0.524	1.34E-04	2.75E-04
tetracycline__tet(34)	0.792	2.10E-11	1.00E-10
tetracycline__tet(J)	0.791	2.28E-11	1.04E-10
tetracycline__tet(L)	0.750	8.21E-10	2.71E-09
tetracycline__tet(S)	0.688	6.44E-08	1.92E-07
trimethoprim__dfrE	0.653	4.79E-07	1.21E-06
trimethoprim__dfrG	0.630	1.64E-06	3.82E-06
vancomycin__vanTC	0.515	1.79E-04	3.60E-04
